# IGFBP‐4 enhances VEGF‐induced angiogenesis in a mouse model of myocardial infarction

**DOI:** 10.1111/jcmm.15516

**Published:** 2020-06-28

**Authors:** Da Wo, Jinxiao Chen, Qiongyu Li, En Ma, Hongwei Yan, Jun Peng, Weidong Zhu, Yong Fang, Dan‐ni Ren

**Affiliations:** ^1^ Department of Plastic and Reconstructive Surgery Ninth People's Hospital Shanghai Jiaotong University School of Medicine Shanghai China; ^2^ Fujian Key Laboratory of Integrative Medicine on Geriatric Academy of Integrative Medicine Fujian University of Traditional Chinese Medicine Fuzhou China; ^3^ Key Laboratory of Arrhythmias of Ministry of Education Clinical and Translational Research Center Research Institute of Heart Failure Shanghai East Hospital Tongji University School of Medicine Shanghai China

**Keywords:** angiogenesis, angiopoietin‐1, IGFBP‐4, myocardial infarction, oxidative damage, Vascular endothelial growth factor

## Abstract

Vascular endothelial growth factor (VEGF) is a well‐known angiogenic factor, however its ability in promoting therapeutic angiogenesis following myocardial infarction (MI) is limited. Here, we aimed to investigate whether dual treatment with insulin‐like growth factor binding protein‐4 (IGFBP‐4), an agent that protects against early oxidative damage, can be effective in enhancing the therapeutic effect of VEGF following MI. Combined treatment with IGFBP‐4 enhanced VEGF‐induced angiogenesis and prevented cell damage via enhancing the expression of a key angiogenic factor angiopoietin‐1. Dual treatment with the two agents synergistically decreased cardiac fibrosis markers collagen‐I and collagen‐III following MI. Importantly, while the protective action of IGFBP‐4 occurs at an early stage of ischemic injury, the action of VEGF occurs at a later stage, at the onset angiogenesis. Our findings demonstrate that VEGF treatment alone is often not enough to protect against oxidative stress and promote post‐ischemic angiogenesis, whereas the combined treatment with IGFBP4 and VEGF can utilize the dual roles of these agents to effectively protect against ischemic and oxidative injury, and promote angiogenesis. These findings provide important insights into the roles of these agents in the clinical setting, and suggest new strategies in the treatment of ischemic heart disease.

## 1 INTRODUCTION

Myocardial infarction (MI) is associated with the rapid occurrence of oxidative stress‐induced DNA damage.^1^, ^2^, ^3^ Recently, we discovered that insulin‐like growth factor‐binding protein‐4 (IGFBP‐4) can robustly protect against MI via limiting early‐stage DNA damage occurrence and ischaemic injury.^4^

Vascular endothelial growth factor (VEGF) is a well‐known angiogenic factor.^5^ Direct administration of VEGF protein has been shown to enhance blood vessel flow and improve cardiac function.^6^, ^7^, ^8^ However, delivery of VEGF to the injured myocardium following myocardial infarction (MI) often has limited efficacy.^9^, ^10^ MI causes widespread irreversible myocardial damage; thus, the angiogenic effect of VEGF is limited to promoting vascularization around the peri‐infarct zone. Because the cardioprotective action of IGFBP‐4 occurs early on following ischaemic injury, we examined whether combined IGFBP‐4/VEGF therapy can utilize the dual roles of these agents to enhance the effect of VEGF following MI.

## 2 METHODS

### 2.1 Mouse model of MI

10‐week‐old male C57BL/6 mice were anaesthetized by intraperitoneal administration with sodium pentobarbital. Left anterior descending artery was ligated using 7‐0 polypropylene sutures and subsequently, PBS, purified mouse proteins of VEGF (5 μg, Sino Biological) and/or IGFBP‐4 (10 μg, GenScript) was immediately injected using micro‐syringe (Hamilton) at 3 sites surrounding the peri‐infarct border of infarcted myocardium. For longer time point studies, thoracotomy and intracardiac injections of proteins were reperformed at 1 week following MI.

### 2.2 Histological sectioning

Hearts were fixed, paraffin‐embedded, sectioned (6 μM) and stained with Masson's trichrome. Infarct size was determined according to the ratio of infarcted tissue versus total endocardial circumference.

### 2.3 Two‐dimensional echocardiography

Two‐dimensional echocardiography was performed with the Vevo 2100 Imaging System (VisualSonics). Mice were anaesthetized using 1% isoflurane (Sigma‐Aldrich) supplemented with oxygen.

### 2.4 Cell culture and assays

HUVEC was cultured using DMEM with 10% FBS. CCK8 viability assay (Beyotime Biotechnology) was performed by measuring absorbance at 450 nm. Cells were transfected using RNAiMAX (Invitrogen) with siRNAs for human angiopoietin‐1 or negative control in Opti‐MEM. Final cell culture concentrations of vehicles were 1 μg/mL for IGFBP‐4, 0.5 μg/mL for VEGF and 100 μM for H_2_O_2_.

### 2.5 Matrigel analysis

HUVEC was cultured on growth factor–reduced Matrigel (BD Biosciences), and treated with PBS, IGFBP‐4 and/or VEGF for 12 hours, and H_2_O_2_ to examine vascular capillary structures.

### 2.6 Western blot

Total proteins were extracted using RIPA buffer containing protease/phosphatase inhibitors. Membranes were incubated with primary antibodies for: angiopoietin‐1 (ProteinTech), γ‐H2AX, GAPDH, and HRP‐conjugated secondary antibodies (Cell Signaling Technology).

### 2.7 Real‐time PCR

Real‐time PCR was performed with SYBR Green (Takara) using an ABI Prism 7500HT instrument (Applied Biosystems). GAPDH was used as internal control.

### 2.8 Statistical Analysis

Statistical analyses were performed with SPSS 17.0 software. Results were presented as mean ± SEM. Student's *t* test or one‐way ANOVA was used to compare differences between two groups, or more, respectively. *P* < .05 was considered statistically significant.

## 3 RESULTS

### 3.1 Dual IGFBP‐4/VEGF treatment decreases fibrosis following MI in mice

**Figure 1:**
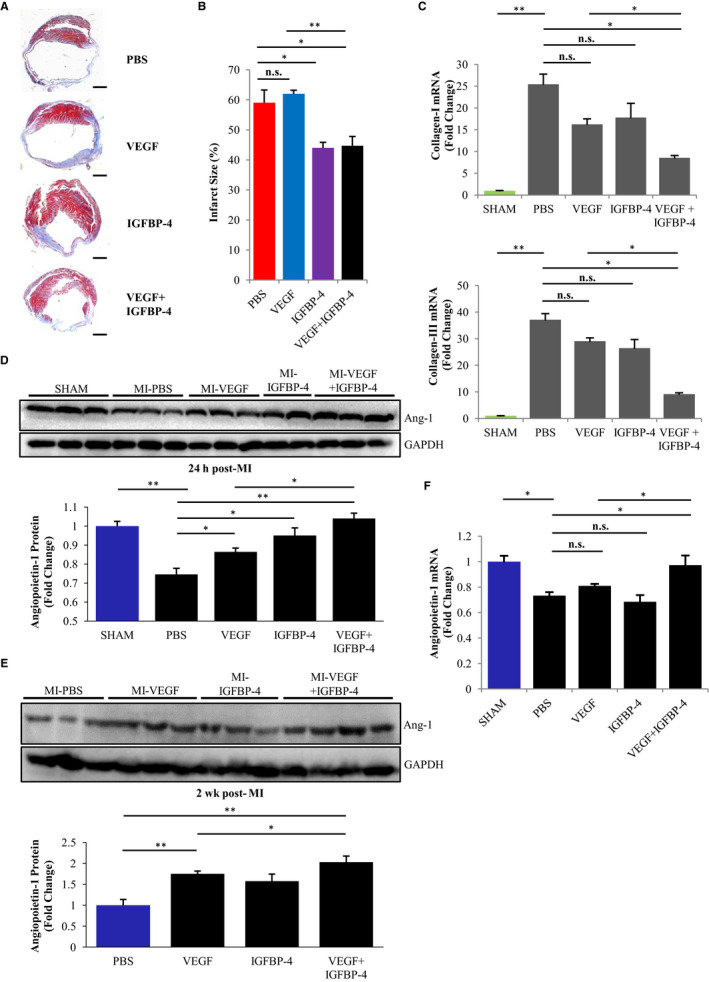
Dual IGFBP‐4/VEGF treatment decreases fibrosis and promotes angiogenesis in mice following myocardial infarction. A, Representative Masson's trichrome–stained sections of hearts at 4 wk post‐myocardial infarction following treatment with PBS, IGFBP‐4 and/or VEGF. Red, muscle fibres. Blue, collagen‐stained areas of fibrosis. Scale bar, 1 mm. B, Quantification of infarct size measurements of hearts based on the region of fibrosis as a percentage of heart endocardial circumference. n = 5 for each. *P < .05, **P < .01; n.s., no significance. C, Real‐time PCR analysis showing mRNA expressions of fibrosis markers collagen I and collagen III at 2 wk post‐myocardial infarction in the peri‐infarct border zone of hearts following treatment with PBS, IGFBP‐4 and/or VEGF. SHAM group was set as fold change of 1. n = 5 for each. *P < .05, **P < .0001; n.s., no significance. D, Immunoblots and quantification of protein expressions of pro‐angiogenic factor angiopoietin‐1 at 24 h post‐myocardial infarction in hearts following treatment with PBS, IGFBP‐4 and/or VEGF. n = 4 for each. GAPDH, internal control. *P < .05, **P < .01. E, Immunoblots and quantification of protein expressions of pro‐angiogenic factor angiopoietin‐1 at 2 wk post‐myocardial infarction in hearts following treatment with PBS, IGFBP‐4 and/or VEGF. n = 4 for each. GAPDH, internal control. *P < .05, **P < .01. F, Real‐time PCR analysis showing mRNA expressions of pro‐angiogenic factor angiopoietin‐1 at 2 wk post‐myocardial infarction in the peri‐infarct border zone of hearts following treatment with PBS, IGFBP‐4 and/or VEGF. SHAM group was set as fold change of 1. n = 5 for each. **P < .05; n.s., no significance

We performed mouse models of MI and intracardiac injections of PBS, VEGF and/or IGFBP‐4 following ischaemia. At 4 weeks post‐MI, Masson's trichrome staining showed no significant improvements in infarct size in VEGF‐treated mice compared with PBS‐treated mice (Figure 1A), but distinct reductions in IGFBP‐4–treated and dual VEGF/IGFBP‐4–treated mice (Figure 1B), indicative of decreased degree of cardiac fibrosis. Closer analysis of the peri‐infarct border showed that dual treatment of VEGF/IGFBP‐4 resulted in significant and synergistic decreases in the expressions of fibrosis markers collagen I and collagen III following MI (Figure 1C), which can be attributed to the enhanced angiogenic action of VEGF, but only when the ischaemic myocardium is not too damaged beyond repair such as following IGFBP‐4 treatment.

Echocardiography showed VEGF treatment alone and had no significant improvements in ejection fraction and stroke volume (Figure S1A), or LV wall motion (Figure S1B) compared with PBS‐treated mice; however, dual IGFBP‐4/VEGF treatment significantly improved these parameters. These results indicated that VEGF treatment alone offered no significant improvements to cardiac function following MI, but was cardioprotective when used in combination with IGFBP‐4.

### 3.2 Combined IGFBP‐4/VEGF treatment promotes angiogenesis via activating pro‐angiogenic angiopoietin‐1 in vivo

We examined the protein expression of angiopoietin‐1, a mediator of angiogenesis and vascular integrity, which was rapidly and significantly down‐regulated in the peri‐infarct border of infarcted hearts 24 hours post‐MI compared with SHAM‐operated mice. Although the treatment with VEGF or IGFBP‐4 alone somewhat attenuated angiopoietin‐1 down‐regulation, dual IGFBP‐4/VEGF‐treated mice completely prevented down‐regulation of angiopoietin‐1 expression (Figure 1D). Similarly, at 2 weeks post‐MI, the expression of angiopoietin‐1 was highest in dual IGFBP‐4/VEGF treatment hearts, significantly higher than PBS‐treated hearts (Figure 1E). Furthermore, mRNA expression of angiopoietin‐1 was significantly decreased in PBS‐, VEGF‐ or IGFBP‐4–treated hearts, but was virtually restored in combined IGFBP‐4/VEGF treatment hearts (Figure 1F). These results indicate a combined cardioprotective effects of dual VEGF/IGFBP‐4 treatment against ischaemic injury via increasing the expression of angiopoietin‐1.

### 3.3 IGFBP‐4 protects against H_2_O_2_‐induced cell damage/death in HUVEC

**Figure 2:**
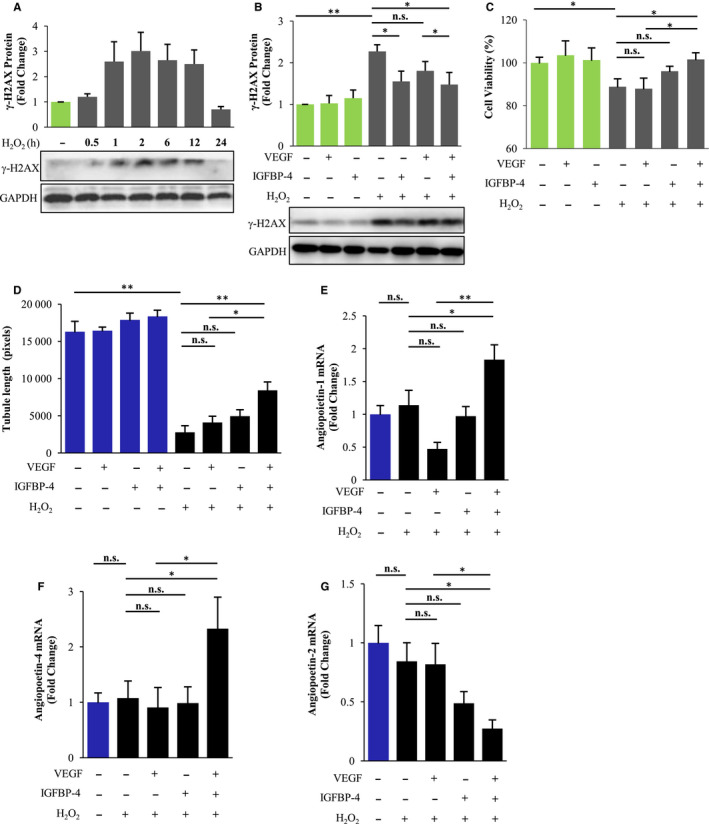
Combined IGFBP‐4/VEGF treatment enhances cell viability, vascularization and expression of pro‐angiogenic factors following H_2_O_2_ treatment in HUVEC. A, Immunoblots showing the time course of H_2_O_2_ treatment that can induce rapid DNA damage, as indicated by the expression of γ‐H2AX in HUVEC. GAPDH, loading control. Representative images of three separate experiments. B, Immunoblots and quantification of total protein expressions of DNA damage marker γ‐H2AX, following IGFBP‐4 and/or VEGF pre‐treatment and subsequent H_2_O_2_ treatment in HUVEC. GAPDH, internal control. Representative images of three separate experiments. *P < .05, **P < .01; n.s., no significance. C, CCK8 assay of cell viability in HUVEC following pre‐treatments of IGFBP‐4 and/or VEGF, prior to H_2_O_2_ treatment for 1 h. n = 7 for each. *P < .05; n.s., no significance. D, Quantification of Matrigel‐based tube formation in HUVEC following pre‐treatments of IGFBP‐4 and/or VEGF, prior to H_2_O_2_ treatment for 1 h. n = 6 for each. *P < .01, **P < .0001; n.s., no significance. E‐G, Real‐time PCR analysis showing mRNA expressions of pro‐angiogenic factors angiopoietin‐1 (E) and angiopoietin‐4 (F), as well as anti‐angiogenic factor angiopoietin‐2 (G), following IGFBP‐4 and/or VEGF pre‐treatment and H_2_O_2_ treatment in HUVEC. n = 4 for each. *P < .05, **P < .01; n.s., no significance

Hydrogen peroxide (H_2_O_2_) treatment is widely used to mimic oxidative stress‐induced damage, which rapidly and significantly activated expression of γ‐H2AX, an early marker of DNA damage in HUVEC (Figure 2A), but was significantly attenuated by dual pre‐treatments with VEGF/IGFBP‐4 (Figure 2B and Figure S2A). H_2_O_2_ treatment also significantly decreased HUVEC cell viability, which was significantly attenuated by combined pre‐treatment using VEGF/IGFBP‐4 (Figure 2C). These results demonstrate that combined IGFBP‐4/VEGF treatment can protect against H_2_O_2_‐induced oxidative damage and DNA damage occurrence in HUVEC.

### 3.4 IGFBP‐4 enhances VEGF ability to promote angiogenesis in vitro

Increased proliferation of endothelial cells in response to external stress factors commonly signifies the occurrence of angiogenesis. In the absence of external stress, there were no significant differences in Matrigel tube formation of three‐dimensional capillary‐like structures in HUVEC, but was significantly decreased following H_2_O_2_ treatment. IGFBP‐4‐ or VEGF‐treated cells also exhibited few such formations, but cells treated with both IGFBP‐4/VEGF exhibited significant and large areas of tube structure formation following H_2_O_2_ treatment, demonstrating that IGFBP‐4 enhances the ability of VEGF for angiogenesis under oxidative stress (Figure 2D). Thus, dual IGFBP‐4/VEGF treatment pre‐conditioned cells to withstand prolonged oxidative damage, thereby promoting vascularization.

Furthermore, dual VEGF/IGFBP‐4 treatment significantly up‐regulated the mRNA expression of angiopoietin‐1 following H_2_O_2_ treatment (Figure 2E), and another angiogenic family member angiopoietin‐4 (Figure 2F), but significantly decreased the expression of anti‐angiogenic angiopoietin‐2 (Figure 2G). Notably, siRNA knockdown of angiopoietin‐1 blocked the effect of IGFBP‐4 and VEGF in protecting against H_2_O_2_‐induced oxidative damage (Figure S2A) and cell death (Figure S2B). Taken together, these results suggest that IGFBP‐4 enhances the effect of VEGF via activating the expression of pro‐angiogenic angiopoietins.

## 4 DISCUSSION

Vascular endothelial growth factor and a separate angiogenic factor angiopoietin‐1 play complementary roles during angiogenesis.^11^, ^12^ However, angiogenesis is closely related to the degree of oxidative stress, where proper mitigation has a positive effect in promoting angiogenesis and tissue repair.^13^ Therefore, it is likely that the treatment of VEGF protein promotes angiogenesis via regulating the expression of angiopoietins only when the degree of oxidative stress is small and manageable, such as during dual treatment with IGFBP‐4.

The synergistic angiogenic effect of IGFBP‐4/VEGF suggests the likelihood that IGFBP‐4 may also improve the efficacy of other angiogenic factors, which requires further investigation. Because IGFBP‐4 is a robust cardioprotective agent against cardiac ischaemic injury,^4^ combined therapy of IGFBP‐4 with other angiogenic factors may be a promising strategy for the treatment of MI.

Thus, our findings demonstrate that VEGF treatment alone is limited in promoting angiogenesis and cardioprotection following MI, whereas dual treatment with IGFBP‐4 can not only enhance VEGF‐induced angiogenesis, but also effectively protect against ischaemic and oxidative injury. These findings provide important insights into the roles of these agents in the clinical setting and suggest new strategies in the treatment of ischaemic heart disease.

## CONFLICTS OF INTEREST

None.

## AUTHOR CONTRIBUTIONS

**Da Wo:** Conceptualization (equal); Data curation (equal); Formal analysis (lead); Funding acquisition (supporting); Investigation (lead); Methodology (equal); Visualization (lead); Writing–original draft (lead); Writing–review & editing (equal). **Jinxiao Chen:** Data curation (equal); Formal analysis (lead); Investigation (equal); Methodology (equal); Resources (equal); Visualization (equal). **Qiongyu Li:** Data curation (equal); Formal analysis (equal); Investigation (equal); Methodology (equal); Resources (equal); Validation (lead); Visualization (supporting). **En Ma:** Investigation (supporting); Methodology (supporting). **Hongwei Yan:** Methodology (supporting); Resources (supporting); Software (supporting). **Jun Peng:** Project administration (supporting); Resources (equal); Software (equal). **Weidong Zhu:** Conceptualization (equal); Funding acquisition (equal); Project administration (equal); Resources (supporting). **Yong Fang:** Investigation (equal); Project administration (lead); Resources (lead); Software (equal); Supervision (equal). **Dan‐ni Ren:** Conceptualization (lead); Funding acquisition (lead); Project administration (lead); Resources (equal); Software (equal); Supervision (equal); Writing–review & editing (equal).

## Supporting information

Figure S1‐S2

## Data Availability

The data that support the findings of this study are available from the corresponding author upon reasonable request.
