# Supplementary figures and images for: IGFBP‐4 enhances VEGF‐induced angiogenesis in a mouse model of myocardial infarction
Source: J Cell Mol Med. 2020 Jun 28;24(16):9466–71. doi: 10.1111/jcmm.15516 (PMC7417680; doi:10.1111/jcmm.15516)

Supplementary Figure 1

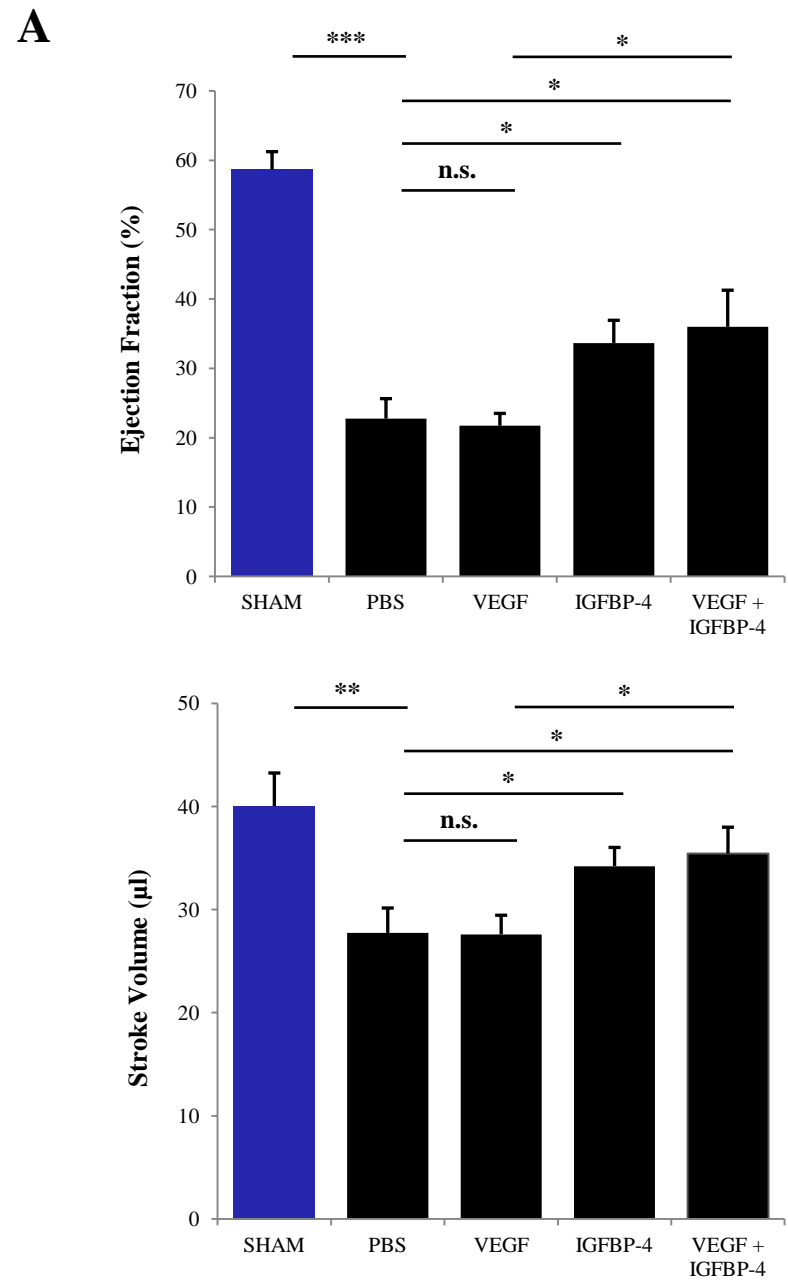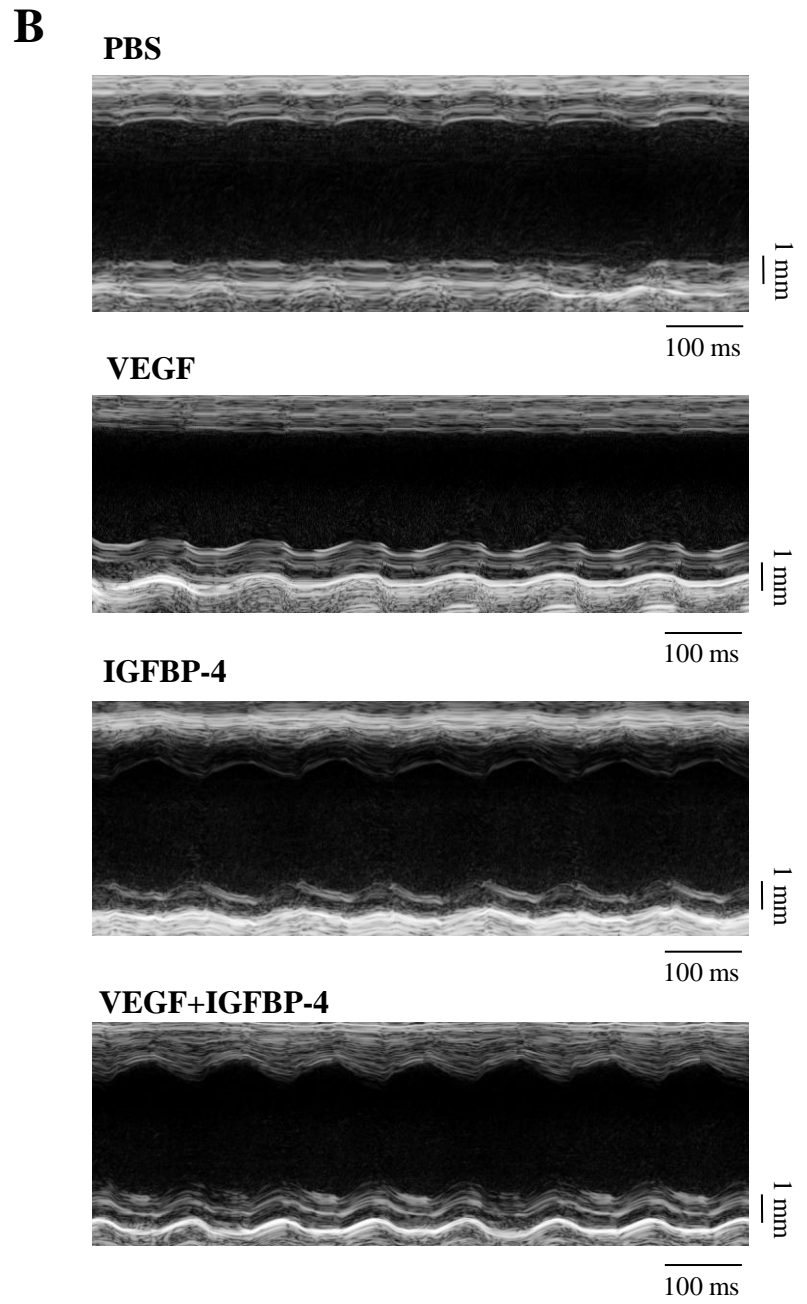

Supplementary Figure 2

A

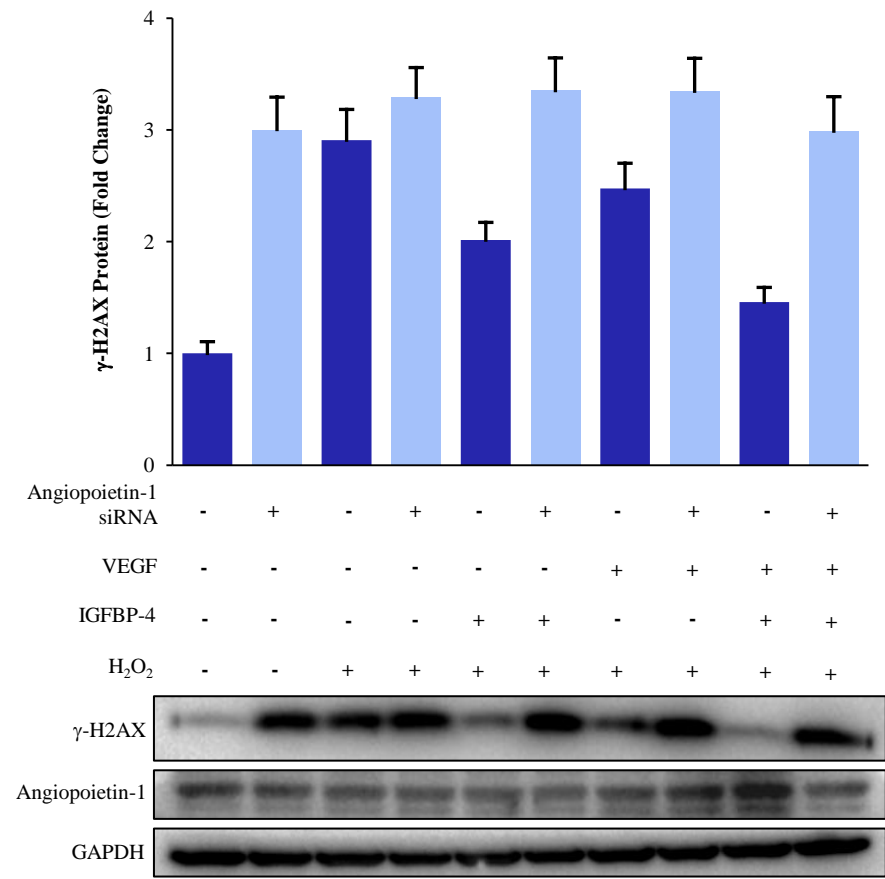

B

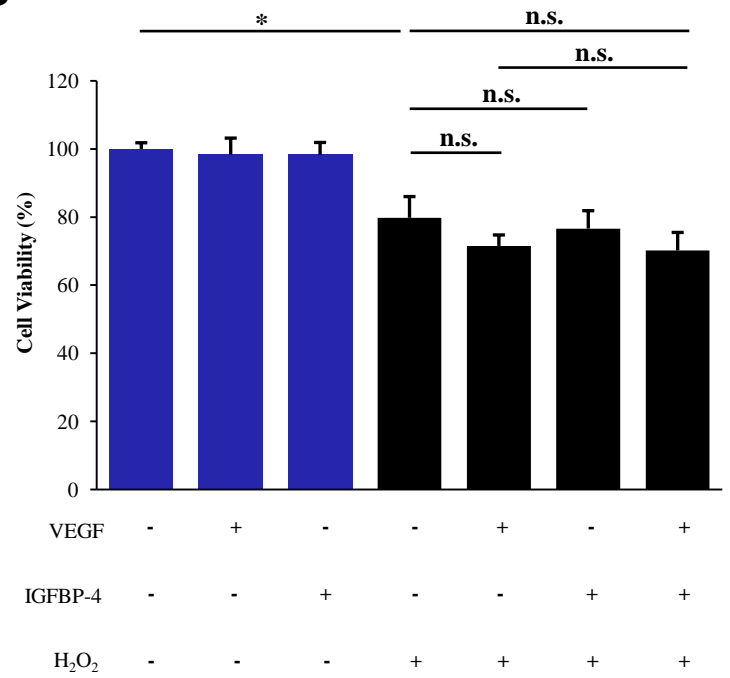

Supplement: Supplementary file 1 — Figure S1‐S2 [file JCMM-24-9466-s001.pdf]
